# An integrative approach to phylogeny reveals patterns of environmental distribution and novel evolutionary relationships in a major group of ciliates

**DOI:** 10.1038/srep21695

**Published:** 2016-02-16

**Authors:** Ping Sun, John Clamp, Dapeng Xu, Bangqin Huang, Mann Kyoon Shin

**Affiliations:** 1 Key Laboratory of the Ministry of Education for Coastal and Wetland Ecosystem, College of the Environment and Ecology, Xiamen University, Xiamen 361102, China; 2 Fujian Province Key Laboratory for Coastal Ecology and Environmental studies, Xiamen University, Xiamen 361102, China; 3 Department of Biology, North Carolina Central University, Durham, NC 27707, USA; 4 State Key Laboratory of Marine Environmental Science, College of Ocean and Earth Sciences, Xiamen University, Xiamen 361102, China; 5 Department of Biological Sciences, University of Ulsan, Ulsan 680-749, Korea

## Abstract

Peritrichs are a major group of ciliates with worldwide distribution. Yet, its internal phylogeny remains unresolved owing to limited sampling. Additionally, ecological distributions of peritrichs are poorly known. We performed substantially expanded phylogenetic analyses of peritrichs that incorporated SSU rDNA sequences of samples collected from three continents, revealing a number of new relationships between and within major lineages that greatly challenged the classic view of the group. Interrogation of a dataset comprising new environmental sequences from an estuary and the open ocean generated with high throughput sequencing and clone libraries plus putative environmental peritrich sequences at Genbank, produced a comprehensive tree of peritrichs from a variety of habitats and revealed unique ecological distribution patterns of several lineages for the first time. Also, evidence of adaptation to extreme environments in the Astylozoidae clade greatly broadened the phylogenetic range of peritrichs capable of living in extreme environments. Reconstruction of ancestral states revealed that peritrichs may have transitioned repeatedly from freshwater to brackish/marine/hypersaline environments. This work establishes a phylogenetic framework for more mature investigations of peritrichs in the future, and the approach used here provides a model of how to elucidate evolution in the context of ecological niches in any lineage of microbial eukaryotes.

Ciliates are a large group of microbial eukaryotes encompassing ~8000 described species^1^. Peritrichs, which are found worldwide in a broad array of marine and freshwater environments, are some of the most abundant ciliates and are heavily involved in nutrient cycling owing to their prodigious ability as suspension feeders^2^,^3^,^4^,^5^.

Peritrich ciliates, excluding mobilid ciliates, generally are accepted as a subclass within class Oligohymenophorea. They are characterized morphologically by mode of development of the feeding stage (solitary vs. colonial), structure of the stalk/lorica, infraciliature of the oral area, and pattern of the silverline system^6^. The group comprises 14 families and 105 genera, with over 800 species described so far^7^. Recent molecular evidence indicates that the Peritrichia is monophyletic. However, it is still difficult to determine relationships among/within families and genera of peritrichs because relatively few representatives have been sequenced in most of them. Moreover, the coverage is rather uneven, focusing mainly on the single, large genus *Vorticella*^8^,^9^,^10^,^11^,^12^,^13^,^14^,^15^,^16^.

Despite the widespread presence of peritrichs in nature, there is still relatively little understanding of their ecological characteristics because of under-sampling and sampling bias. A handful of free-living species that inhabit permanent bodies of fresh water have been studied intensively over the years as indicators of water quality in natural ecosystems and sewage treatment systems^4^. By contrast, the vast majority of peritrichs, especially ones in ectosymbiotic relationships or that occur in estuaries, offshore and extreme environments—have received much less attention. Molecular advances, such as clone libraries and high-throughput sequencing, have made sequencing of samples from diverse environments possible and contribute huge numbers of environmental sequences to public databases, which are a great resource for exploring ecologically and phylogenetically related issues^17^,^18^,^19^,^20^,^21^,^22^. Therefore, we hypothesized that interrogating new datasets generated by these methods from environments which are rarely represented in previous studies, combined with publicly available sequences with clear taxonomic designations and previously collected environmental sequences for peritrichs in Genbank, would permit a better assessment of the extent of the environmental diversity of peritrichs and their distribution among different environments^23^,^24^.

The aims of the present study, therefore, were (i) to clarify the internal relationships of the subclass Peritrichia by increasing taxon sampling from a variety of habitats across large geographic regions; and (ii) to reveal the ecological distribution of taxa in a phylogenetic context based on environmental peritrich datasets encompassing a) new environmental data generated from samples collected from previous rarely represented environments, b) newly obtained, nearly full length sequences, and c) sequences with clear taxonomic designations as well as a wide array of environmental sequences from Genbank; and (iii) to describe a group I intron found in three populations of *Vorticella gracilis* collected from two geographically distant regions (China and Austria) that represents the first discovery of its type in ciliates.

## Results

### Phylogenetic analysis

**Figure 1:**
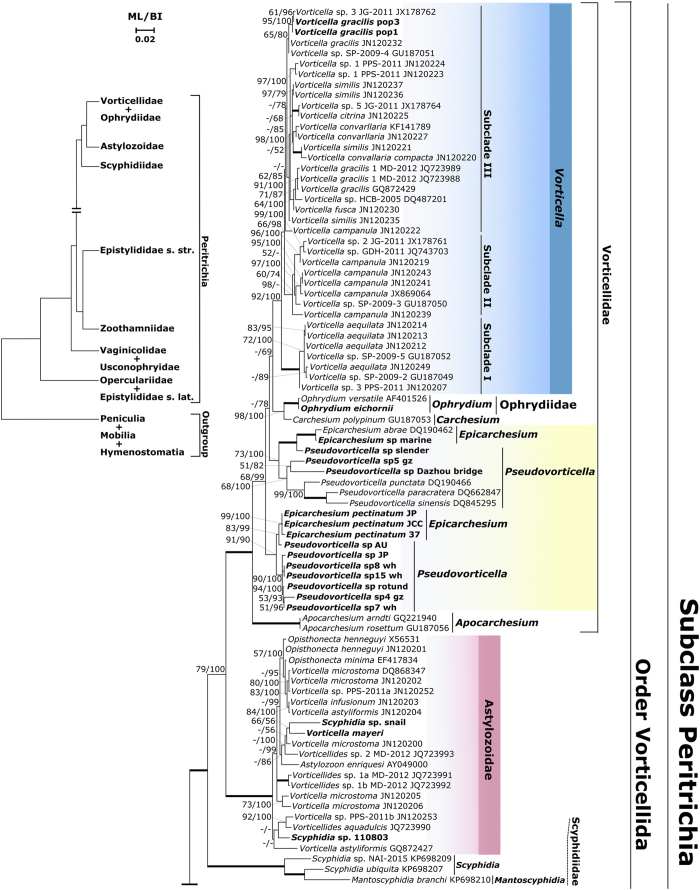
The upper part of maximum likelihood (ML) tree of peritrichs with support values from maximum likelihood and Bayesian inferences (BI) on/under the internal branches. The figure is split at a major node into (1) and (2) to allow the tree to be presented clearly. Thick branches denote maximal support values. Newly sequenced taxa are in boldface.

The phylogenies derived from SSU rDNA sequences with maximum likelihood (ML) and Bayesian inference (BI) methods were generally congruent with each other and, thus, were combined into a consensus tree for the following descriptions. Altogether, sequences of nine currently recognized families were included in phylogenetic analyses, which yielded support for a variety of previously unconfirmed or unsuspected relationships between and within taxa (Fig. 1).

In the family Vorticellidae, an association of species of *Ophrydium* (family Ophrydiidae) with *Carchesium polypinum* was highly supported in both trees. Both *Pseudovorticella* and *Epicarchesium* were paraphyletic, with two sessile, marine species of *Epicarchesium* grouped with species of *Pseudovorticella* in one of two sister clades and the free-swimming, freshwater species *E*. *pectinatum* in the other one (Fig. 1).

Monophyly of the clade containing species of *Vorticellides*, *Astylozoon*, *Scyphidia*, and *Opisthonecta* was supported with maximum values. *Scyphidia* sp. from a freshwater snail clustered with *V*. *mayeri* instead of its morphological congener *S.* sp11010803, which was clearly separated from a small cluster of the family Scyphidiidae containing three species symbiotic on patellogastropods (Fig. 1).

**Figure 2:**
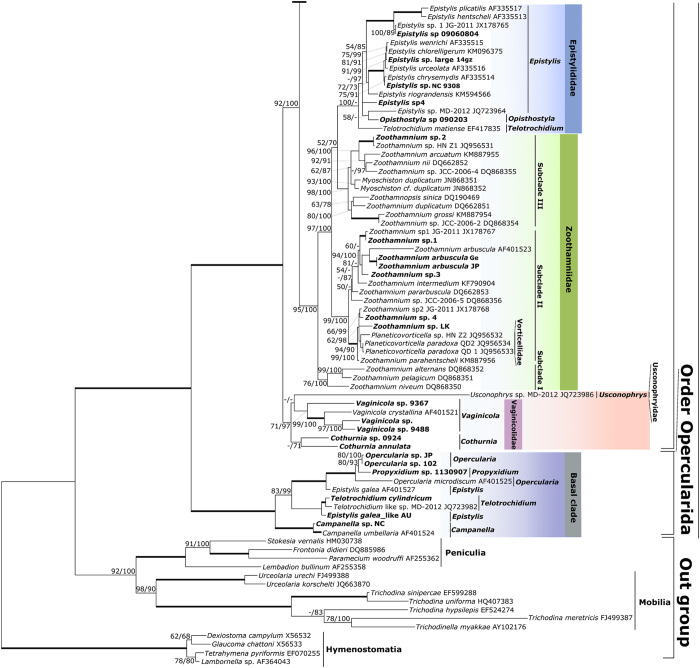
The lower part of maximum likelihood (ML) tree of peritrichs with support values from maximum likelihood and Bayesian inferences (BI) on/under the internal branches. The figure is split at a major node into (1) and (2) to allow the tree to be presented clearly. Thick branches denote maximal support values. Newly sequenced taxa are in boldface.

The family Zoothamniidae comprised three genetic clusters (Fig. 2). Subclade I containing three species of *Zoothamnium* was basal to the other two. Subclade II, including species of *Zoothamnium* and *Planeticovorticella* (family Vorticellidae), was sister to the clade consisting of subclade III and Epistylididae. Subclade III consisting of species of *Zoothamnium*, *Zoothamnopsis* and *Myoschiston* was sister to a large clade containing the majority of species in the Epistylididae.

The family Epistylididae consisted of four genetic clusters (Fig. 2). The largest one, which included the majority of species of *Epistylis*, was nested within the clades comprising the family Zoothamniidae^8^,^9^,^10^,^12^,^16^. This clade consisted of two relatively large subclades and a poorly resolved association between *Epistylis* sp4, *Epistylis* sp. MD-2012, and *Opisthostyla* sp. The secondarily free-swimming species *Telotrochidium matiense* (family Opisthonectidae) occupied a separate divergent branch that was basal to the entire epistylidid clade.

The other three clusters of epistylidids fell into the basal clade of peritrichs (=order Opercularida^16^) with full support. Two sequences of *Campanella* spp. occupied the basal position in the clade. *Epistylis galea* grouped with species of *Opercularia* and *Propyxidium* (family Operculariidae), and a species of *Epistylis* resembling *E*. *galea* clustered with two species of *Telotrochidium*. Monophyly of the family Operculariidae was fully supported, with a single species of *Propyxidium* nested within *Opercularia* spp. (Fig. 2).

A clade consisting of species in the loricate families Vaginicolidae and Usconophryidae occupied the basal position in the crown clade of peritrichs (=order Vorticellida^16^). In this clade, two species of *Cothurnia* were sister to *Usconophrys* sp. and a well-supported cluster comprised of four species of *Vaginicola* (Fig. 2).

### Environmental distribution pattern inferred from environmental phylogeny

**Figure 3:**
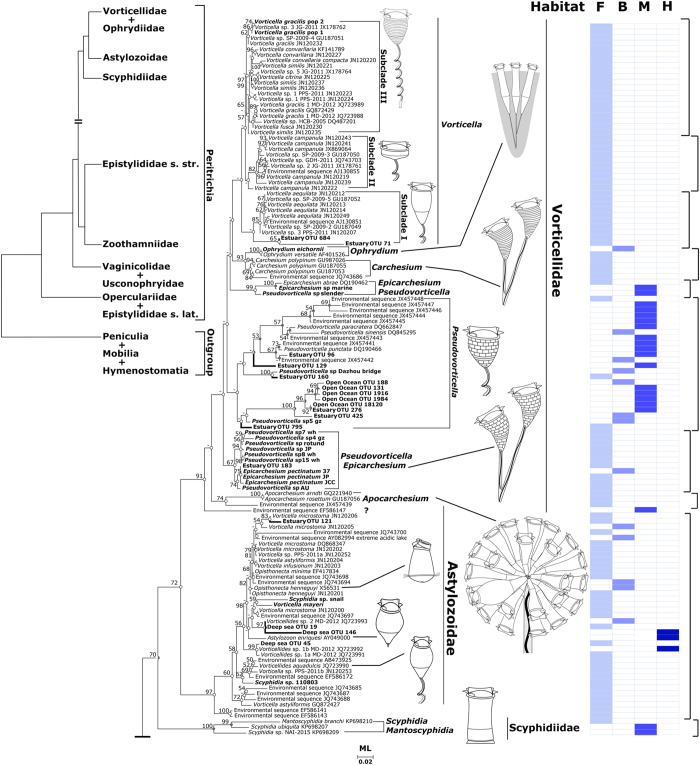
The upper part of maximum likelihood (ML) phylogeny of peritrich SSU rDNA sequences from the present study and candidate sequences with or without taxonomic designations from Genbank. Ancestor states were parsimoniously inferred: white circle, freshwater; light gray circle, brackish; gray circle, marine; and black circle, hypersaline. Insets, salinities (F, freshwater; B, brackish; M, marine; H, hypersaline) and diagrammatic representations of key genera of peritrichs.

**Figure 4:**
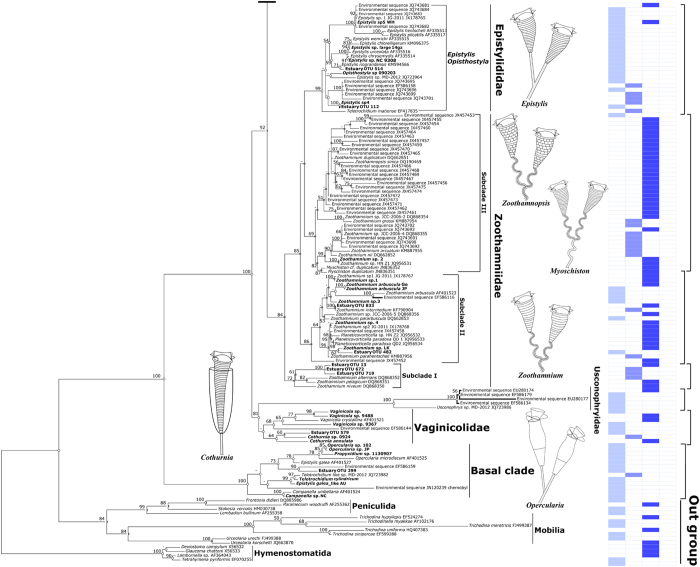
The lower part of maximum likelihood (ML) phylogeny of peritrich SSU rDNA sequences from the present study and candidate sequences with or without taxonomic designations from Genbank. Ancestor states were parsimoniously inferred: white circle, freshwater; light gray circle, brackish; gray circle, marine; and black circle, hypersaline. Insets, salinities (F, freshwater; B, brackish; M, marine; H, hypersaline) and diagrammatic representations of key genera of peritrichs.

We investigated the environmental distribution of peritrichs based on sequences obtained in this study and sequences with/without clear taxonomic designations from GenBank. In the environmental tree (Fig. 3), the Vorticellidae clade was represented by sequences that were mostly from freshwater habitats whereas the Astylozoidae clade was highly diverse, with its sequences mapping to a wide variety of freshwater, estuarine, marine, and hypersaline habitats, e.g. freshwater lakes^25^, bamboo stumps in a neotropical area^26^, rivers and coastal areas^27^,^28^,^29^, an extremely acidic lake with a high concentration of heavy metal^30^, ponds, ephemeral pools, puddles^13^, an estuary, the mantle cavity of snails, the body surface of an amphipod (present study) and deep hypersaline anoxic basins^19^. A high proportion of environmental sequences mapped to the family Zoothamniidae, with sequences of all clusters except subclade II coming from brackish/marine habitats only. In contrast to zoothamniids, most sequences of the family Epistylididae were from freshwater habitats. The basal clade (order Opercularida) was uniquely characterized by having all sequences from a variety of freshwater habitats except one sequence from an estuary with a salinity of 3.6‰ (Fig. 4).

### Identification, predicted secondary structure and phylogenetic analyses of introns

Introns that were 402, 408, and 410 nt in length were identified in the SSU rDNA sequences of three populations of *Vorticella gracilis*. The introns of populations 1 (408 nt) and 2 (410 nt), collected in Austria, were identical except for two insertions of adenine at positions 636 and 660 in the sequence of population 2. The intron of population 3 (402 nt), collected in China, contained a 6 nt deletion compared with that of population 2. According to the reference SSU rDNA sequence of *Escherichia coli* (accession number J01695), the intron positions were determined to be at nt. 242 for three populations of *V. gracilis*, thus named as Vgr.S242 following the nomenclature suggested by^31^.

**Figure 5:**
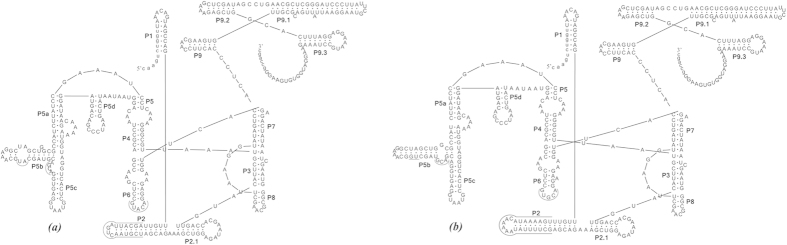
Secondary structure of *Vorticella gracilis* group IC1 intron. (**a**) Vgr.S242 in *V. gracilis* pop1. (**b**) Vgr.S242 in *V. gracilis* pop3. The intron and exon sequences are given in uppercase and lowercase characters, respectively.

The secondary structures of introns of three populations of *V. gracilis* were predicted and the differences between the structures of populations 1 and 3 are underlined in Fig. 5. The predicted secondary structures of populations 1 and 2 were identical except for two nucleotides. Therefore, the secondary structure of the latter is not shown. The predicted secondary structures consisted of nine base-paired segments and conserved elements were characterized by comparison with the structures. Based on similarity of sequence and secondary structure, the three group I introns were inferred to be members of subgroup IC1, and no open reading frame of significant size was identified.

**Figure 6:**
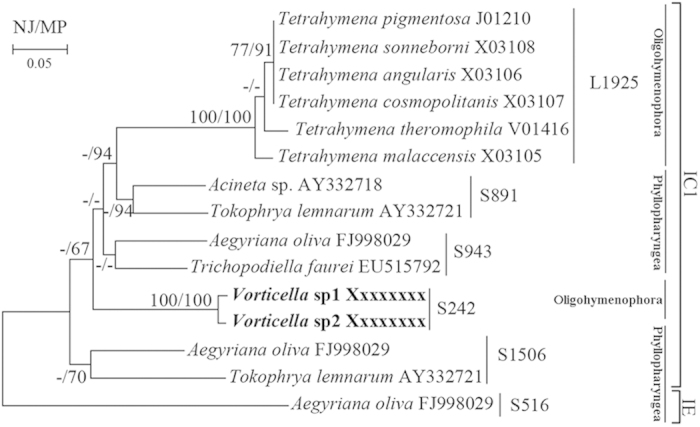
Phylogeny of group I introns of ciliates. Neighbor Joining (NJ) and Maximum Parsimony (MP) bootstrap values over 50 are shown on branch. The intron insertion sites in both large and small ribosomal DNA are shown on the right side.

Sequences of previously reported introns of ciliates were collected from GISSD and Genbank and used to construct an intron tree of ciliates. In the tree, sequences were recovered as two divergent clades, subgroups IC1 and IE (Fig. 6). Within the IC1 clade, sequences from the same insertion position predictably grouped as clusters although bootstrap values for many nodes were low.

## Discussion

Altogether, 147 SSU rDNA sequences of peritrichs from this study and Genbank were included in our phylogenetic analyses. This data set yielded a number of new insights about relationships among and within several families of peritrichs.

The family Vorticellidae Ehrenberg, 1838 is the largest taxon in subclass Peritrichia. Originally, vorticellids were differentiated morphologically from other peritrichs by possession of a helically contractile spasmoneme within the stalk (Fig. 3, insets), and historically, it was considered to be a well-defined group based on this characteristic alone^1^,^3^. Previous phylogenetic investigations showed that *Ophryidium versatile* (family Ophryidiidae) clustered with vorticellids, contrary to morphological evidence^10^,^16^. However, this association was considered doubtful because only a single sequence of ophrydiids was available, and their morphology is so unlike that of vorticellids (see inset in Fig. 3). Therefore, the sequence has been omitted from some previous analyses^9^,^13^. Addition of two sequences of *O*. *eichornii* to our analyses confirmed a close relationship to one species of *Carchesium* (Fig. 1). Also, an AU Test supported monophyly of vorticellids with *Ophrydiium* included (Table S1).

The affinity between *Ophrydium* and *Carchesium* is unexpected because, at first glance, the former does not resemble *Carchesium* at all (rigid stalk, gelatinous lorica in *Ophrydium* vs. helically contractile stalk, no lorica in *Carchesium*; see insets in Fig. 3). However, they do resemble one another with respect to some basic morphological characteristics common to most members of the crown clade of peritrichs (=order Vorticellida), such as being colonial, possessing an eversible peristomial lip, and having a transverse rather than reticulate silverline system. The position of *Ophrydium*, nested deeply within family Vorticellidae suggests that it represents a lineage that acquired its rigid stalk by reduction and loss of the spasmoneme in its ancestor and that its gelatinous lorica was either convergently evolved with respect to the loricae of vaginicolids or an evolutionary novelty.

Species of *Pseudovorticella* and *Epicarchesium* are distinguished from other members of the family Vorticellidae by possession of a reticulate silverline system^32^ (Fig. 3, insets). In previous analyses, three sequences of *Pseudovorticella* (solitary species) formed a single cluster with *E. abrae* (colonial form)^10^,^12^. Addition of ten sequences of *Pseudovorticella*, one sequence of *Epicarchesium*, and three sequences of *E*. *pectinatum* (colonial, free-swimming) revealed that both genera are non-monophyletic (Figs 1 and 2a). *Epicarchesium pectinatum* is clustered within one cluster of *Pseudovorticella* species, indicating that it is not congeneric with other species of *Epicarchesium*. It may represent a unique planktonic form that has acquired the ability to form colonies as a means of dispersing itself. Mature colonies of *E*. *pectinatum* fragment to produce new colonies^33^ and never have been observed to produce telotrochs or alternate with an attached form (Clamp, pers. obs.). Considering its molecular phylogenetic position and distinctive morphology and mode of development, it is likely that *E*. *pectinatum* represents a new genus. However, this hypothesis needs to be tested by addition of more sequences of *Pseudovorticella* in its clade. Additionally, inclusion of environmental sequences revealed that a single sequence (EF586147) was on a separate branch that was basal to the entire Vorticellidae with strong support, indicating that it might represent the tip of a branch in a much bigger tree because many species of the family Vorticellidae remain to be sequenced (Fig. 3).

A previous study confirmed that some morphospecies of *Vorticella* (family Vorticellidae) actually are members of family Astylozoidae and redefined both families on the basis of molecular characters^13^. The redefined Astylozoidae contains an exceptionally diverse set of morphospecies, including ones formerly placed in the Vorticellidae (genus *Vorticellides*) and Opisthonectidae (genus *Opisthonecta*) in addition to those that were originally assigned to the Astylozoidae (genus *Astylozoon*). In the present study, newly obtained sequences of *Vorticella mayeri*, which is a free-swimming planktonic vorticellid and two ectosymbiotic species in the family Scyphidiidae all fell within the Astylozoidae clade which was clearly separated from A small genetic cluster consisting of three symbiotic species of scyphidiidae, suggesting that the family Scyphidiidae is polyphyletic and contains at least some species that should be reassigned to the Astylozoidae (Fig. 1). This also expanded morphological diversity within Astylozoidae, most of which derives from evolutionary loss of developmental stages (e.g., loss of telotroch stage in *Astylozoon*; loss of trophont stage in *Opisthonecta*). The newly identified members of the Astylozoidae clade certainly conform to this pattern. *Vorticella mayeri* appears to lack a telotroch stage^3^, and trophonts of *Scyphidia* spp. have lost the ability to form a stalk, attaching to a host with a thin layer of material secreted by the scopula^1^. In addition, inclusion of sixteen environmental sequences of unidentified astylozoid taxa in the environmental phylogeny revealed that more members of the Astylozoidae clade await formal discovery (Fig. 3).

The type species of *Planeticovorticella* has a complex life cycle that includes a trophont with the helically contractile stalk characteristic of vorticellids^34^. Therefore, it was surprising that *P*. *paradoxa* clustered with species of *Zoothamnium* in subclade II (Fig. 2). However, this species was assigned to *Planeticovorticella* on the basis of a similarity in development and appears to lack a stage with a contractile stalk^35^. Moreover, it is an ectosymbiont of bivalve molluscs rather than free-living like the type species, *P*. *finleyi*. Consequently, it is likely that the developmental similarity between the two species is the result of convergent evolution rather than descent from a common ancestor. Unfortunately, *P. finleyi* has not yet been sequenced to provide a means to identify with confidence the genus to which *P. paradoxa* belongs.

*Opisthonecta*, type genus of the family Opisthonectidae Foissner, 1975, was reassigned to the family Astylozoidae by^13^. However, *Telotrochidium matiense*, another opisthonectid (permanently in telotroch phase of life cycle, never developing trophonts), clustered with the major clade of epistylidids^10^. In our trees, *Telotrochidium* was polyphyletic, with *T*. *matiense* basal to the major Epistylididae clade and others within the basal clade (Fig. 2). Association with epistylidids in a basal position suggests that *T*. *matiense* originated by loss of the trophont stage from its life cycle early in the evolution of the epistylidid major clade rather than, as might be expected, from an ancestor within the clade and that it followed a separate evolutionary pathway thereafter. Association of two morphospecies of *Telotrochidium* with a morphospecies of *Epistylis* in the basal clade strongly confirms the hypothesis that loss of the trophont stage to create a secondarily free-swimming, permanent telotroch has evolved convergently in more than one lineage of peritrichs.

The family Zoothamniidae Stiller, 1951 was established for the large, colonial genus *Zoothamnium* and several small genera with a myoneme in the stalk that contracts in a “zig-zag” conformation (insets to Fig. 3). Previous phylogenetic studies revealed that species of *Zoothamnium* either grouped into a single clade or clustered as unresolved parallel branches, with strong support for terminal nodes within the clade but poor support for deeper ones^9^,^10^. The addition of new sequences in our trees recovered members of Zoothamniidae as three well-supported subclades, suggesting that the family is a paraphyletic assemblage (Fig. 2). In addition to evidence provided by generally strong support values for deeper nodes, an AU test also failed to support monophyly of Zoothamniidae (Table S1).

Polyphyly of epistylidids was strongly supported (Fig. 2). The major clade of epistylidids was nested within Zoothamniidae, casting doubt on the validity of Epistylididae. The previous study^12^ proposed that Epistylididae s. str. might be a distinct, plesiomorphic, basally positioned clade of peritrichs within the crown clade. However, addition of more sequences of both zoothamniids and epistylidids, as well as inclusion of multiple sequences of loricate peritrichs for the first time, reveals that epistylidids may be a group of zoothamniids s. lat. that have experienced secondary loss of the stalk spasmoneme. The developmental anomaly of *Myoschiston* spp., zoothamniids in which the spasmoneme degenerates from basal stalk upward in mature colonies^14^ (inset to Fig. 4, note the truncation of the spasmoneme at the first branching of the colony), supports this hypothesis.

Species in the families Vaginicolidae and Usconophryidae were recovered as an unresolved monophyletic assemblage (Fig. 2), with phylogenetic position of Usconophryidae differing between trees constructed by different methods. However, addition of environmental sequences recovered both vaginicolids and usconophryids as monophyletic groups (Fig. 4). An AU test also did not refute the possibility that Vaginicolidae is monophyletic (Table S1). Furthermore, a close relationship among loricate peritrichs is confirmed in both trees, suggesting that possession of a lorica is a plesiomorphic character in this assemblage. However, loricate peritrichs are a diverse group in their own right, and a large number and variety of taxa have not been sequenced yet. It remains to be seen, then, whether or not they represent a separate, ordinal-level taxon as proposed by Kahl 1935.

Previous studies revealed that the morphological epistylidids *Epistylis galea* and *Campanella umbellaria* fell within the basal clade in a position widely divergent from the major epistylidid clade^16^. Addition of new sequences showed that, as expected, *Campanella* sp. clustered with its congener *C. umbellaria* in the basal clade, but another species of *Epistylis* resembling *E. galea* clustered with two species of *Telotrochidium* with full support instead of with *E*. *galea*, which grouped with members of the Operculariidae (Fig. 2). Like epistylidids, members of Operculariidae have a rigid, non-contractile stalk, but they differ markedly from them (and all other peritrichs) by having a non-eversible peristomial lip (inset to Fig. 4). In our trees, operculariids were recovered as a monophyletic group with maximal support, which confirms the uniqueness of the morphology of their peristomial lip as a diagnostic feature. Aside from operculariids, the basal clade as a whole is well-supported as the monophyletic order Opercularida but, internally, is a poorly resolved group of species with no distinctive morphological characters in common. There are many species of operculariids described in literatures that have not been sequenced yet. If these taxa are found to cluster with members of Operculariidae included in this analysis, the family will be recognized as the dominant taxon in the basal clade. However, it is far from clear whether the remaining members of the basal clade would form a single, as yet unrecognized family or would be revealed as a cluster of small, mostly monotypic families.

In the end, it should be noted that not all sequences submitted to Genbank are taxonomically verified. Therefore, caution should be exercised regarding unexpected phylogenetic placements resulting from inclusion of sequences without any accompanying morphological data.

### Findings inferred from interrogated environmental dataset

We integrated the present set of SSU rDNA/rRNA sequences with available environmental data to form a dataset that comprises the largest number of environmental peritrich sequences compiled to date. It provided a preliminary “snapshot” of the environmental distribution of peritrich taxa that, at least, begins to elucidate their evolution in the essential context of their ecological niches.

In the environmental tree, most sequences of *Vorticella* s. str. were from freshwater, certainly reflecting at least some degree of sampling bias because there are some species of *Vorticella* reported from marine habitats^2^ (Fig. 3). However, this ecological profile might be generally true because it is unlikely that sequences of such easily detected ciliates, given their wide distribution and high abundance in aquatic environments, were overlooked in previous environmental studies^28^,^29^. In previous phylogenetic studies, only three species of *Pseudovorticella* from intertidal areas were available. Addition of 10 new sequences from freshwater and 20 environmental sequences from the open ocean, an estuary, coastal areas, and freshwater revealed that most of brackish/marine sequences of *Pseudovorticella* fell within the less basally positioned of the three genetic clusters of *Pseudovorticella*. This suggests that the apparent paraphyly of vorticellids with a reticulate silverline system might be the result of divergence of a brackish/marine lineage from a freshwater one.

A surprising result was the placement of sequences from marine habitats in the Astylozoidae clade because no marine species of astylozoids have been reported by previous investigators. This indicates that astylozoids have an even more diverse environmental distribution than the wide range of mostly ephemeral habitats from which they are reported^7^,^13^,^26^,^36^. Moreover, the Astylozoidae clade encompassed species that represent an extremely wide range of adaptations, given that their sequences came from a wide variety of extreme environments (e.g., deep hypersaline anoxic basins in the Mediterranean Sea with salinities ranging from 63–348‰; the Rio Tinto, an extremely acidic river with pH of 2 in Spain with high concentration of heavy metals; soils in Spain polluted by polycyclic aromatic hydrocarbon for over 90 years^19^,^30^,^37^). The presence of astylozoids in these extreme environments suggests that they may possess the ability to cross a wide range of environmental gradients, which uniquely distinguishes them from other peritrichs because many protists are known to have relatively narrow ranges of salinity/pH/pollutant tolerance^38^. This result also broadens the phylogenetic range of ciliates that are capable of living in extreme environments.

The clades of zoothamniids consisted primarily of marine species with subclades I and III coming from brackish/marine environments only (Fig. 4). A small cluster of sequences from the freshwater species *Z*. *arbuscula* together with another two freshwater sequences nested within subclade II, suggesting that at least the free-living species of *Zoothamnium* comprise a series of marine lineages that, with few exceptions, have not penetrated into freshwater habitats. This is not surprising, given that few free-living, freshwater species of *Zoothamnium* have been reported in the literature. However, many epizoic species of *Zoothamnium*, some of which occur on freshwater hosts, are not represented in the dataset, and thus, it cannot be determined yet if they conform to the pattern seen in the environmental tree. In contrast to *Zoothamnium*, species in the major clade of *Epistylis* comprised a large freshwater assemblage with a few brackish/marine sequences nested within it, suggesting that colonizations of *Epistylis* into the marine habitat have taken place as well.

The environmental tree is consistent with the historical literature with regard to vaginicolids and taxa in the basal clade (Fig. 4). Vaginicolids are known as free-living and epizoic species in a variety of freshwater and marine habitats. By contrast, the basal clade appears to consist of freshwater forms except one sequence from an estuarine environment with a salinity of 3.6‰. No members of the Operculariidae have been reported from marine or estuarine habitats, and both *Campanella* and *E*. *galea* are known only from freshwater.

To summarize, the overall pattern of distribution revealed by the environmental tree and reconstruction of ancestral states suggest that peritrichs originated in freshwater and diversified there to some extent before giving rise to the crown clade. Evolution within the crown clade appears to have involved both a proliferation of major body plans and adaptive radiation into an extremely wide variety of habitats. Zoothamniids appear to represent a paraphyletic “grade rather than a clade” that has evolved mainly in marine environments, with some few taxa (e.g. *Z*. *arbuscula*) having recolonized freshwater (Fig. 4). Epistylidids may represent an offshoot of zoothamniids characterized not only by loss of the spasmoneme but by secondary reinvasion of freshwater habitats. Vorticellids already have been revealed as a morphological ‘grade'^13^ but, in contrast to zoothamniids, appear to have diversified mainly in freshwater with relatively few transitions into brackish/marine habitats.

It should be noted that relatively few species could be included in the environmental tree compared to the total of more than 800 species of peritrichs reported in the taxonomic literature (Table S2). Possible reasons for this may be the following: (i) many previous environmental investigations have focused on planktonic species and, thus, would have detected relatively few peritrichs other than ones attached to plankton^28^; (ii) only one study used peritrich-specific primers whereas other environmental diversity studies employed eukaryotic/ciliate-specific primers that recovered only sequences of species that either were abundant in the environment investigated or possessed high rDNA copy numbers^39^; and (iii) many peritrichs that are ectosymbionts on zooplankton and large algae were not detected because most environmental diversity studies have focused on micro- or even smaller-sized organisms by filtering water samples to remove organisms larger than 200 μm.

### Group I intron and a ciliate intron tree

Group I introns have been found in a number of major eukaryotic lineages (e.g. fungi, plants, protists), but their distribution is highly scattered because most eukaryotic species appear to lack them. Group I introns have been classified into five major groups (IA-IE) and fourteen subgroups (IA1-3, IB1-4, IC1-3, ID, IE1-3) based on comparative sequence analysis^40^. In ciliates, thirteen intron sequences altogether have been reported in two classes, Oligohymenophorea and Phyllopharyngea. The group I intron described here in three populations of *Vorticella gracilis* was the first discovery of its type in ciliates. The ciliate intron tree (Fig. 6) that was constructed based on the new sequences and all other available ones revealed two findings: (i) group I intron sequences grouped according to their subgroup as well as the insertion positions without regard to the phylogenetic relationship of their hosts (e.g., *Aegyriana oliva* hosted three subtypes of introns—Aol.S516, Aol.S943, Aol.S1506—that clearly clustered with other ciliate intron sequences at the same insertion position rather than grouping together), and (ii) introns from three populations of *V. gracilis* collected from geographically distant places were closely similar nonetheless in their primary sequences and secondary structures, suggesting that the intron originated from an ancient vertical transfer into the SSU rDNA sequence. Relatively few intron sequences of ciliates were available compared to the great number of species (8000+) reported. Therefore, introns must be discovered in many more taxa to reveal their true evolutionary history in ciliates.

## Conclusions

The ultimate function of phylogeny and taxonomy is to lay out a pathway for stepwise investigation of integrative roles of organisms in nature. Unfortunately, these disciplines have too often been treated as an end in themselves. Peritrichs and other ciliated protists represent a large proportion of biomass in inverse proportion to their microscopic size and are now known to comprise a key part of many ecosystems, with their participation in “microbial loops” perhaps the most universally recognized example^41^. The present phylogeny revealed widespread genealogical non-monophyly in families and genera of peritrichs. This demonstrates that the current catalogue of ciliate genera/families is incomplete and suggests that a large scale multitaxa study from different geographical regions and types of habitats will be useful for reconstructing and testing evolutionary relationships of ciliates. The environmental phylogeny of peritrichs that was constructed in this study also stands as an example of how the pattern of ecological distribution holds the potential for revealing hidden diversity and understanding evolutionary history of any given group of microbial eukaryotes. Particularly, being able to address two questions in a coherent, productive way, (i) how are specific morphological adaptations related to their ecological niches? and (ii) what specific molecular adaptations are characteristic of individual lineages and how are they related to coping with specific environmental demands?, is ample justification for making the effort necessary to elucidate the phylogenetic and evolutionary relationships of any group of microeukaryotes.

## Material and Methods

### Collection, isolation, fixation, and identification of organisms

Forty-nine strains of peritrichs were collected from a wide variety of aquatic environments—shorelines, streams, bogs, lakes, ephemeral habitats, mariculture/aquaculture ponds, and ponds in botanical gardens in Asia, Europe and North America (Table S3). Isolation, fixation and identification of peritrichs followed^15^.

Ecological distribution of peritrichs was investigated by collecting samples from two environments, the estuary of the Jiulong River in Xiamen, Fujian, China and the East Tropical Pacific Ocean off the western coast of South America, which were under-represented in previous studies. Eleven estuarine sites (117°80′–118°13′E, 24°36′–24°46′N) were sampled across the entire salinity gradient. For each sample, 2 L-3 L of surface and subsurface waters were pre-filtered with a 200 μm sieve to remove mesozooplankton and then directly filtered through a polycarbonate filter of 3 μm pore size (Millipore), followed by storage in RNAlater (Qiagen, Germany). Approximately 2 L of surface water was collected from three sites (83°3′W, 2°27′S; 89°28′W, 2°45′S; 86°0′W, 2°44′S) in the East Tropical Pacific Ocean, then were filtered through a 3 μm polycarbonate filter, followed by storage in liquid nitrogen for further treatment.

Terminology and taxonomic classification used in the current paper largely follow^1^. Following^42^, all sessile peritrichs are taken as Peritrichia. Also, we followed^16^ in accepting the orders Vorticellida and Opercularida.

### DNA/RNA extraction, pcr amplification, Sanger and high throughput sequencing

DNA extraction, PCR amplification and sequencing followed^13^. Sequence fragments were assembled into contiguous sequences and edited with the Sequencher 4.0 software package (GeneCodes Corp., Ann Arbor, MI).

Genomic DNA of East Tropical Pacific Ocean samples was extracted using PowerWater DNA isolation Kit (Mo Bio Laboratories, Inc.). Clone libraries were constructed using primers specific for ciliates^26^, then sequenced by the Sanger method. Assembly of sequence fragments was done as above.

Extraction of RNA, reverse-transcription and nested pcr for estuarine samples followed^19^ except different pairs of ciliate specific primers^43^ were used for the first PCR. Purified amplicons were sequenced by Majorbio (Shanghai, China) using the Illumina MiSeq platform.

### Alignments and molecular phylogenetic analyses

Genbank accession numbers of newly obtained sequences are shown in Table S4. For sequences with 100% identity derived from different samples, only one was included in analyses. In order to avoid loss of important informative sites, only nearly complete sequences of peritrichs available from GenBank were included in strictly phylogenetic analyses, and their accession numbers are shown in Fig. 1. Sequences were aligned using HMMER, version 2.1.4 with default settings and taking secondary structure into account^44^. Then the alignment was edited using Bioedit version 7.0.9^45^.

Lengths and GC content of sequences are given in Table S4. Altogether, 147 sequences with 1533 unambiguously aligned sites were included in alignments for expanded phylogenetic analyses. Fifteen sequences of the subclasses Hymenostomatia, Mobilia and Peniculia were added to phylogenetic analyses as outgroups.

The GTR + I + G model was selected for Maximum likelihood (ML) analyses by jModeltest 2^46^. An ML tree was constructed with RAxML using the GTR + gamma model because this was the best fitting one available in RAxML on CIPRES Sciences Gateway^47^, and support for the best-scoring ML tree was assessed by 1000 bootstrap (BP) replicates. Bayesian analysis was performed with MrBayes 3.1.2^48^ using the GTR + I + G model selected by MrModeltest 2^49^ under the AIC criterion. Four simultaneous Markov chain Monte Carlo (MCMC) chains were run for 6,000,000 generations, sampling every 100 generations. The first 25% of trees were discarded as burn-in. The 50% majority rule consensus tree was used to calculate the posterior probabilities (PP) for each node. Trees were viewed and edited with MEGA V4.0^50^.

### Analyses of environmental OTUs

Short reads generated by high throughput sequencing were filtered for quality with QIIME^51^ employing several criteria. Reads that had quality scores lower than 20, more than two primer base mismatches, and were shorter than 300 bp were removed. OTUs were picked using Uparse at the 97% sequence similarity threshold^52^. Generation of an OTU table and taxonomic assignment of each OTU were done with Qiime using the reference taxonomic database of ciliates from the Protist ribosomal reference database (PR2)^53^. Sequences generated by clone libraries were clustered into OTUs and then processed to taxonomic assignment using the same method as above. The environmental peritrich amplicons generated in this study were deposited in Genbank (Table S2).

### Interrogating environmental sequences

First, we interrogated nearly full-length environmental SSU rDNA sequences of peritrichs deposited in Genbank to detect environmental distribution patterns of species. Short sequences (<1400 bp) were filtered out from 1490 putative sequences of peritrichs, and duplicated sequences were de-replicated, leaving 60 environmental sequences from seven previously published studies of freshwater planktonic communities^25^,^27^,^35^, coastal planktonic communities^28^,^29^, biofilms from Chernobyl exposed to sunlight and gamma radiation^54^, and extremely acidic water with high concentrations of heavy metals from the Rio Tinto^30^.

Sequences of peritrichs obtained with ciliate-specific primers from habitats that have not been represented in previous investigations, deep hypersaline anoxic basins (DHAB) in the Mediterranean Sea^19^, and biofilms of streams in Auckland, New Zealand^42^ were added to analyses. Environmental peritrich OTUs from DHAB samples were generated using QIIME. Biofilm sequences were blasted against the NCBI nr database to reveal putative peritrich sequences and their accession numbers are given in Table S2.

Altogether, 40 short environmental peritrich sequences (19 from the Xiamen estuary, 3 from DHAB, 5 from the East Tropical Pacific Ocean and 13 from New Zealand biofilms) were appended to a dataset that included 147 sequences with clear taxonomic designations and 60 nearly full-length environmental sequences. Each short sequence was then aligned to its best blast hit using the pairwise alignment option in Bioedit and verified by visual examination. An ML tree was constructed with RAxML, and alternative phylogenetic placements of short sequences were estimated by EPA using likelihood weights. Placements with support of ≥95% were considered to have high confidence, and those with <95% low confidence^55^. Finally, the most parsimonious pattern of freshwater-brackish/marine/hypersaline transitions was inferred for peritrichs using MESQUITE 2.75^56^. According to the Venice Salinity Classification System, character states were classified as 1 = Freshwater (<0.5 PSU), 2 = Brackish(0.5–30 PSU), 3 = Marine(30–40 PSU), and 4 = Hypersaline (>40 PSU).

## Additional Information

**How to cite this article**: Sun, P. *et al.* An integrative approach to phylogeny reveals patterns of environmental distribution and novel evolutionary relationships in a major group of ciliates. *Sci. Rep.* **6**, 21695; doi: 10.1038/srep21695 (2016).

## Supplementary Material

Supplementary Information
