# Supplementary material for: An integrative approach to phylogeny reveals patterns of environmental distribution and novel evolutionary relationships in a major group of ciliates
Source: Sci Rep. 2016 Feb 16;6:21695. doi: 10.1038/srep21695 (PMC4754944; doi:10.1038/srep21695)
Supplement: Supplementary Information [file srep21695-s1.pdf]

An integrative approach to phylogeny reveals patterns of environmental distribution and novel evolutionary relationships in a major group of ciliates

Ping Sun[1,2,*], John Clamp[3], Dapeng Xu[4,*], Bangqin Huang[1], Mann Kyoon Shin[5]

[1]Key Laboratory of the Ministry of Education for Coastal and Wetland Ecosystem, College of the Environment and Ecology, Xiamen University, Xiamen 361102, China
[2]Fujian Province Key Laboratory for Coastal Ecology and Environmental studies, , Xiamen University, Xiamen 361102, China
[3]Department of Biology, North Carolina Central University, Durham, NC 27707, USA
[4]State Key Laboratory of Marine Environmental Science, College of Ocean and Earth Sciences, Xiamen University, Xiamen 361102, China
[5]Department of Biological Sciences, University of Ulsan, Ulsan 680-749, Korea

[*]Correspondence and requests for materials should be addressed to P.S. (psun@xmu.edu.cn) and D.X. (dapengxu@xmu.edu.cn)

Table S1. Approximately unbiased test (AU) results based on SSUrDNA data.

Hypothesis tested	AU
Monophyly of Vorticellidae+Ophryidiidae	0.576
Monophyly of Astylozooidae+Scyphidiaspp.+Vorticella mayeri	0.297
Monophyly of Zoothamniidae	0.029
Monophyly of Epistylis s. str.	0.302
Monophyly of Vaginicolidae	0.303
Monophyly of Scyphididae	8e-005

Table S2.Information of peritrich OTUs/environmental sequences that were included in the present study. BW, brackish water; DHAB, Deep hypersaline anoxic basins, Mediterranean Sea; ETP, Surface water, Eastern Tropical Pacific; FW, freshwater; GR, Guangdang River, Yantai, China; IMGS, Îles de la Madeleine, Gulf of St. Lawrence, Canada; JR, Estuary of Jiulong River, Fujian, China; LG2012, Liu and Gong 2012; M, marine; PS, present study; S2013, Stock et al. 2013; T2012, Toupoint et al. 2012.

Name/Accession Number	Sample Location	Salinity	NtSource		Data Source
			DNA	RNA	
Estuary_OTU13 KU363293	JR	3‰		X	PS
Estuary_OTU71 KU363289	JR	0.7‰		X	PS
Estuary_OTU96 KU363288	JR	0.7‰		X	PS
Estuary_OTU112 KU363285	JR	0.5‰		X	PS
Estuary_OTU121 KU363297	JR	0.5‰		X	PS
Estuary_OTU129 KU363294	JR	15.2‰		X	PS
Estuary_OTU160 KU363296	JR	13.4‰		X	PS
Estuary_OTU183 KU363307	JR	0.9‰		X	PS
Estuary_OTU276 KU363300	JR	3.8‰		X	PS
Estuary_OTU299 KU363287	JR	3.6‰		X	PS
Estuary_OTU425 KU363290	JR	29.8‰		X	PS
Estuary_OTU482 KU363308	JR	15.2‰		X	PS
Estuary_OTU514 KU363286	JR	0‰		X	PS
Estuary_OTU579 KU363299	JR	12.2‰		X	PS
Estuary_OTU672 KU363292	JR	25.3‰		X	PS
Estuary_OTU684 KU363298	JR	0.4‰		X	PS
Estuary_OTU719 KU363291	JR	25.3‰		X	PS
Estuary_OTU795 KU363295	JR	0‰		X	PS
Estuary_OTU833 KU363306	JR	3‰		X	PS
Open Ocean_OTU188 KU363302	ETP	M	X		PS
Open Ocean_OTU131 KU363301	ETP	M	X		PS
Open Ocean_OTU1916 KU363304	ETP	M	X		PS
Open Ocean_OTU1984 KU363305	ETP	M	X		PS
Open Ocean_OTU18120 KU363303	ETP	M	X		PS
Deep Ocean_OTU19	DHAB	63-348‰		X	S2013
Deep Ocean_OTU45	DHAB	63-348‰		X	S2013
Deep Ocean_OTU146	DHAB	63-348‰		X	S2013
AJ130855	Lake Ketelmeer, The Netherlands	FW	X		Van-Hannen et al. 1999
AJ130851	Lake Ketelmeer, The Netherlands	FW	X		Van-Hannen et al. 1999
AY082994	Rio Tinto, La Palma del Condado, Spain	FW	X		Amaral-Zettler et al. 2002
AB473925	River Toess, Switzerland	FW	X		Horath et al. 2008
HQ219427	Lakes Pavin and Aydat, France	FW	X		Monchy et al. 2011
JN020239	Biofilms, Chernobyl area, Ukraine	FW	X		Ragon et al. 2011
JQ743680	GR	FW	X		LG2012
JQ743681	GR	FW	X		LG2012
JQ743682	GR	FW	X		LG2012
JQ743683	GR	FW	X		LG2012
JQ743684	GR	FW	X		LG2012
JQ743685	GR	FW	X		LG2012
JQ743686	GR	FW	X		LG2012
JQ743687	GR	FW	X		LG2012
JQ743688	GR	FW	X		LG2012
JQ743690	GR	BW	X		LG2012
JQ743691	GR	BW	X		LG2012

JQ743692	GR	BW	X	LG2012
JQ743693	GR	BW	X	LG2012
JQ743694	GR	BW	X	LG2012
JQ743695	GR	BW	X	LG2012
JQ743696	GR	BW	X	LG2012
JQ743697	GR	BW	X	LG2012
JQ743698	GR	BW	X	LG2012
JQ743699	GR	BW	X	LG2012
JQ743700	GR	BW	X	LG2012
JQ743701	GR	BW	X	LG2012
JQ743702	GR	M	X	LG2012
JX457439	IMGS	M	X	T2012
JX457441	IMGS	M	X	T2012
JX457442	IMGS	M	X	T2012
JX457443	IMGS	M	X	T2012
JX457444	IMGS	M	X	T2012
JX457445	IMGS	M	X	T2012
JX457446	IMGS	M	X	T2012
JX457447	IMGS	M	X	T2012
JX457448	IMGS	M	X	T2012
JX457452	IMGS	M	X	T2012
JX457453	IMGS	M	X	T2012
JX457454	IMGS	M	X	T2012
JX457455	IMGS	M	X	T2012
JX457456	IMGS	M	X	T2012
JX457457	IMGS	M	X	T2012
JX457458	IMGS	M	X	T2012
JX457459	IMGS	M	X	T2012
JX457460	IMGS	M	X	T2012
JX457461	IMGS	M	X	T2012
JX457462	IMGS	M	X	T2012
JX457463	IMGS	M	X	T2012
JX457464	IMGS	M	X	T2012
JX457465	IMGS	M	X	T2012
JX457466	IMGS	M	X	T2012
JX457467	IMGS	M	X	T2012
JX457468	IMGS	M	X	T2012
JX457469	IMGS	M	X	T2012
JX457470	IMGS	M	X	T2012
JX457471	IMGS	M	X	T2012
JX457472	IMGS	M	X	T2012
JX457473	IMGS	M	X	T2012
JX457474	IMGS	M	X	T2012
JX457475	IMGS	M	X	T2012

Table S3.Taxa sampled in the present study.

Taxon	Localities	Date
Campanella sp. NC	United States, NC, Beaufort Co., Durham Creek at Tunstall Swamp Rd.	08/13/2008
Cothurnia annulata	Germany,Höglwörth, Höglwörther See, on stalk of *Zoothamniumarbuscula*	07/27/2009
Cothurnia sp. 0924	China, Qingdao, Zhanqiao Pier, low tide, surface of algae	05/17/2009
Epicarchesium sp. marine	China, Yantai, beach front north of Yantai University, on surface of red algae	06/05/2010
Epicarchesium pectinatum JP	Japan, KarasumaPenisula, Lake Biwa	04/23/2009
Epicarchesium pectinatum JCC	China, S Guangzhou, 1.1 km SSW Shibi, canal at Dazhou Bridge	06/13/2009
Epicarchesium pectinatum 37	China, Guangzhou, South China Botanical Garden, pond	06/10/2009
Epistylis galea-like AU	Austria, Mattsee, marshy area along margin of Mattsee	07/12/2009
Epistylis sp5 pop2 WH	China, Wuhan, park on northwest shore of Donghu lake	05/30/2009
Epistylis sp5pop1 09060804	United States, NC, trail near Duke University, pond	09/06/2008
*Epistylis*sp5pop3 JP	Japan, KarasumaPenisula, Lake Biwa	07/31/2009
Epistylis sp. large 14gz	China, Guangzhou, pond at eastern end of South China Normal University	06/08/2009
Epistylis sp. NC 9308	United States, NC, Orange Co., 10.05 km NW Carrboro center, Drainage channel alongside Dairyland Rd (SR 1177) adjacent to pasture of Mapleview Farms	09/03/2008
*Epistylis*sp4	United States, NC, Cartaret Co., Morehead city, urban pond	08/11/2008
Opercularia sp. JP	Japan, Karasuma Penisula, Lake Biwa	04/2010
Opercularia sp. 102	Germany,Höglwörth, HöglwörtherSee,on stem of dead plant from lake margin	07/27/2009
Ophrydiumeichornii pop1	China, Guangzhou, South China Botanical Garden, pond	05/16/2010
Ophrydiumeichornii pop2	Japan, Karasuma Penisula, Lake Biwa	07/31/2009
*Opisthostyla*sp. 090203	United States, Guam, treatment plant #2	12/18/2008
Propyxidium sp. 1130907	United States, Chapel Hill, Bolin Cr. Greenway, flooded area along trail	01/13/2009
Pseudovorticella sp. Dazhou bridge	China, Guangzhou, rural area south of Guangzhou, pond next to canal at Dazhou bridge	06/13/2009
Pseudovorticella sp5 gz	China, Guangzhou, rural area south of Guangzhou,pond next to canal at Dazhou bridge	06/13/2009
Pseudovorticella sp. slender	United States, North Carolina, Beaufort Co., Goose Creek State Park	10/20/2009
Pseudovorticella sp. AU	Austria, Seekirche, stream, on moss	07/12/2009
Pseudovorticella sp4 gz	China, Guangzhou, South China Botanical Garden, pond	06/10/2009
Pseudovorticella sp7wh	China, Wuhan, marsh exhibit	05/26/2009
Pseudovorticella sp. rotund	United States, Beaufort Co., Goose Creek State Park	10/20/2009
Pseudovorticella sp. JP	Japan, Takashima-shi, Kutsuki. Oisugi	06/15/2009
Pseudovorticella sp15wh	China, Wuhan, park on northwest shore of Donghu lake	05/30/2009
Pseudovorticella sp8wh	China, Wuhan, Huazhong Agricultural University, marsh exhibit	05/26/2009
Scyphidia sp. snail	United States, MA, Lowell	-/-/2009
*Scyphidia*sp. 11010803	United States, NC, trail near Duke University, pond	11/03/2008
Telotrochidium cylindricum	Austria, Guttal, Grossglocknerstrasse, pool in pasture	07/12/2009

Vaginicola sp. pop1 MD	United States, MD, coastal region	11/05/2010
Vaginicola sp. 9488	China, Yantai, on surface of *Enteromorpha* species in mariculture pond	06/08/2010
Vaginicola sp. pop2 CH	China, Xiamen, reservoir at Xiamen University	11/08/2012
Vorticella mayeri	China, Wuhan, fish pond near Sand Lake	06/01/2009
Vorticella gracilis pop1	Austria, S Bruck an der Großglocknerstraße, Wallackhaus area, stream	07/10/2009
Vorticella gracilis pop2	Austria, S Bruck an der Großglocknerstraße, Wallackhaus area, stream	07/10/2009
Vorticella gracilis pop3	China, Xiamen, Furong lake at Xiamen University	11/08/2012
Zoothamnium sp. 1	United States, MD, Shady Side, West River	11/04/2010
Zoothamnium sp. 2	United States, MD, Fairhaven, creek at MD 423	11/04/2010
Zoothamnium sp. 3 pop1	United States, MD, Deale, Rockhold Creek	11/04/2010
Zoothamnium sp. 4	United States, MD, Galesville, West River	11/04/2010
Zoothamnium sp. 3 pop2	United States, MD, Deale, Rockhold Creek	11/05/2010
Zoothamnium arbuscula Ge	Germany,Höglwörth, Höglwörther See, dead leaves in shallow water of lake margin	07/27/2009
Zoothamnium arbuscula JP	Japan, KarasumaPenisula, Lake Biwa	07/21/2010
Zoothamnium sp. 7 pop1 LK	United States, Beaufort Co., Goose Creek State Park	10/20/2009
Zoothamnium sp. 7 pop2	United States, MD, Rhode River	-/-/2008

Table S4.Species for which SSU rRNA sequences were newly sequenced in the present work.

Taxon	GB	SSU rRNA sequence	
		Length (nt)	GC content (%)
Campanella sp. NC	KU363248	1547	41.76
Cothurnia annulata	KU363275	1535	43.91
Cothurnia sp. 0924	KU363268	1535	44.30
Epicarchesium sp. marine	KU363265	1533	43.64
Epicarchesium pectinatum JP	KU363260	1534	43.02
Epicarchesium pectinatum JCC	KU363251	1533	42.92
*Epicarchesium pectinatum*37	KU363246	1534	43.02
Epistylis galea-like AU	KU363242	1551	43.07
Epistylis sp5 pop2 WH	KU363253	1532	43.15
Epistylis sp5 pop1 09060804	KU363256	1532	43.15
Epistylis sp5 pop3 JP	KU363262	1532	43.15
Epistylis sp. large14gz	KU363250	1534	42.89
Epistylis sp.NC 9308	KU363249	1535	43.19
Epistylis sp4	KU363236	1534	42.63
Opercularia sp. JP	KU363267	1549	43.38
Opercularia sp. 102	KU363257	1549	43.25
Ophrydium echornii pop1	KU363264	1534	42.89
Ophrydium echornii pop2	KU363259	1533	42.92
Opisthostyla sp.090203	KU363244	1534	43.22
Propyxidium sp. 1130907	KU363252	1548	43.35
Pseudovorticella sp. Dazhou bridge	KU363283	1533	44.55
Pseudovorticella sp5 gz	KU363277	1533	44.23
Pseudovorticella sp. slender	KU363282	1535	43.71
Pseudovorticella sp. AU	KU363281	1534	43.22
Pseudovorticella sp4 gz	KU363278	1534	43.29
Pseudovorticella sp7wh	KU363279	1534	43.55
Pseudovorticella sp. rotund	KU363284	1534	43.61
Pseudovorticella sp. JP	KU363263	1534	43.61
Pseudovorticella sp15wh	KU363280	1534	43.61
Pseudovorticella sp8wh	KU363276	1535	43.58
Scyphidia sp. snail	KU363254	1535	43.97
*Scyphidia*sp. 11010803	KU363243	1533	43.12
Telotrochidium cylindricum	KU363247	1552	43.23
Vaginicola sp. pop1 MD	KU363269	1535	42.93
Vaginicola sp. 9488	KU363258	1534	42.89
Vaginicola sp. pop2 CH	KU363266	1535	42.87
Vorticella mayeri wh	KU363240	1534	44.00
Vorticella gracilis pop1	KU363238	1553	42.76
Vorticella gracilis pop2	KU363237	1553	42.76
Vorticella gracilis pop3	KU363239	1553	42.82
Zoothamnium sp. 1	KU363270	1534	44.07
*Zoothamnium*s p. 2	KU363271	1534	43.74

Zoothamnium sp. 3 pop1	KU363272	1534	44.39
Zoothamnium sp. 4	KU363273	1532	43.86
Zoothamnium sp. 3 pop2	KU363274	1534	44.39
Zoothamnium arbuscula Ge	KU363245	1534	44.00
Zoothamnium arbuscula JP	KU363261	1536	44.01
Zoothamnium sp. pop1 LK	KU363241	1532	43.67
Zoothamnium sp. pop2	KU363255	1532	43.67
